# Comparative safety and effectiveness of direct oral anticoagulants in patients with atrial fibrillation in clinical practice in Scotland

**DOI:** 10.1111/bcp.13814

**Published:** 2018-12-18

**Authors:** Tanja Mueller, Samantha Alvarez‐Madrazo, Chris Robertson, Olivia Wu, Marion Bennie

**Affiliations:** ^1^ The Farr Institute of Health Informatics Research, Strathclyde Institute of Pharmacy and Biomedical Sciences University of Strathclyde Glasgow UK; ^2^ Department of Mathematics and Statistics University of Strathclyde Glasgow UK; ^3^ Health Protection Scotland NHS National Services Scotland Glasgow UK; ^4^ Institute of Health and Wellbeing University of Glasgow Glasgow UK; ^5^ Public Health and Intelligence Strategic Business Unit NHS National Services Scotland Edinburgh UK

**Keywords:** anticoagulants, atrial fibrillation, clinical effectiveness, DOAC, safety

## Abstract

**Aims:**

The aim of this study was to compare the clinical effectiveness and safety of direct oral anticoagulants (DOACs) in patients with atrial fibrillation (AF) in routine clinical practice.

**Methods:**

This retrospective cohort study used linked administrative data. The study population (*n* = 14 577) included patients with a diagnosis of AF (confirmed in hospital) who initiated DOAC treatment in Scotland between August 2011 and December 2015. Multivariate Cox proportional hazard models were used to estimate hazard ratios of thromboembolic events, mortality and bleeding events.

**Results:**

No differences between the DOACs were observed with regard to the risk of stroke, systemic embolism or cardiovascular death. In contrast, the risk of myocardial infarction was higher among patients prescribed apixaban in comparison to those on rivaroxaban (HR 1.67, 95% CI 1.02‐2.71), and all‐cause mortality was higher among rivaroxaban patients in contrast to both apixaban (1.22 [1.01–1.47]) and dabigatran (1.55 [1.16–2.05]) patients; rivaroxaban patients also had a higher risk of pulmonary embolism than apixaban patients (5.27 [1.79–15.53]). The risk of other major bleeds was higher among rivaroxaban patients compared to apixaban (1.50 [1.10–2.03]) and dabigatran (1.58 [1.01–2.48]) patients; the risks of gastrointestinal bleeds and overall bleeding were higher among rivaroxaban patients than among apixaban patients (1.48 [1.01–2.16] and 1.52 [1.21–1.92], respectively).

**Conclusions:**

All DOACs were similarly effective in preventing strokes and systemic embolisms, while patients being treated with rivaroxaban exhibited the highest bleeding risks. Observed differences in the risks of all‐cause mortality, myocardial infarction and pulmonary embolism warrant further research.

## What is Already Known about this Subject

- Direct oral anticoagulants (DOACs) are increasingly prescribed to patients with atrial fibrillation in order to prevent strokes.
- No clinical trials directly comparing the different DOACs have been conducted, and information regarding the comparative effectiveness and safety of DOACs in clinical practice is still limited.

## What this Study Adds

- This study confirms the comparable effectiveness, but diverging bleeding risk profiles of dabigatran, rivaroxaban and apixaban.
- The study indicates potentially vital differences in the risks of myocardial infarction, pulmonary embolism and mortality among patients treated with individual DOACs.

## Introduction

Atrial fibrillation (AF) is a common arrhythmic disorder particularly among the elderly, and an independent risk factor for stroke – increasing it up to five‐fold 1. Hence, patients with AF are frequently treated long term with oral anticoagulants in order to prevent strokes.

Vitamin K antagonists (VKAs) such as warfarin have been used for this purpose for decades; although bleeding events as a consequence of anticoagulation are not uncommon, VKAs have proven to be sufficiently safe and effective for this indication 2, 3, 4. However, VKA treatment has limitations: it can be inconvenient for patients as it requires constant monitoring; adherence to treatment is considered to be rather low 5; and there are frequently cases, particularly when patients are frail or very elderly, when treatment is not initiated despite the presence of multiple stroke risk factors 6, 7. Therefore, the direct oral anticoagulants (DOACs) dabigatran, rivaroxaban, apixaban and edoxaban have been developed as an alternative means of treatment. Clinical trials, comparing DOACs with warfarin, have proven their safety and efficacy 8, 9, 10, 11; consequently, they have been approved for stroke prevention in patients with AF, and are now increasingly prescribed to patients in many countries, including Scotland.

Nevertheless, although a number of indirect comparisons for the purpose of multiple treatment comparisons of DOACs have been published, including network meta‐analyses facilitating indirect comparisons of multiple treatment groups 12, 13, 14, 15, 16, no clinical trials directly comparing the different DOACs have been conducted; and information regarding the effectiveness and safety of DOACs in clinical practice is still limited. Hence, the purpose of this study was to compare the clinical effectiveness and safety of the different DOACs in patients with AF in routine clinical practice.

## Methods

### Study setting

Scotland has a population of approximately 5.4 million 17, covered by the publicly funded National Health Service (NHS) Scotland. NHS Scotland is divided into 14 regional Health Boards, responsible for planning and delivering services to their respective populations 18, 19. Every resident registered with a general practitioner (GP) in Scotland is issued a Community Health Index (CHI) number, serving as a unique patient identifier throughout the health system 20.

### Study design

The study has been designed as a retrospective cohort study, using routinely collected administrative data: while prescription details originated from the Prescribing Information System (PIS), clinical data were gathered from the Scottish Morbidity Records/Hospital Inpatients and Outpatient attendance datasets (SMR01 and SMR00); date and cause of death have been extracted from National Records of Scotland (NRS). Datasets have been linked via CHI numbers.

PIS was created for payment purposes and combines data about the drugs as well as the prescriber, the dispenser and the costs, for all prescriptions issued and dispensed in the community in Scotland; information relating to drugs is based on the British National Formulary 21, 22. The Scottish Morbidity Records are a series of datasets collecting information about diagnoses and treatment of diseases: SMR01 and SMR00 contain patient‐level data on inpatient/day care discharge episode and appointments in outpatient clinics, respectively. International Classification of Disease codes, 10th edition (ICD‐10) are used in both datasets to record diagnostic details, while Office of Population Censuses and Surveys procedural codes, 4th revision (OPCS‐4) are used to record surgical procedures 23, 24. NRS is the Scottish government department responsible for the registration of life events such as births and deaths; its main data source is the civil registration system 25.

### Study population

The study population comprised patients with AF, either diagnosed or confirmed in secondary care, who started treatment with any DOAC between each drugs' approval date for the prevention of stroke in patients with AF in Scotland (dabigatran: August 2011; rivaroxaban: January 2012; apixaban: January 2013; edoxaban: October 2015) and December 2015. A patient's index date for study inclusion was the date of first recorded prescription for any DOAC. Patients were followed up until the investigated outcome occurred, the patient died or was removed from a Scottish GP register for other reasons, or the study end date (31 December 2015), whichever occurred first. Patients were censored at the time of first treatment discontinuation; time to discontinuation was calculated using the refill‐gap method 26, with an admissible supply gap of 28 days and adjusting for previous oversupply. Censoring for discontinuation included patients switching from their index drug to a different DOAC. Patients where alternative indications for treatment with oral anticoagulants were present (i.e. heart valve replacements, mitral stenosis, or hip/knee replacement surgery or a venous thromboembolism (VTE) during the 6‐month period directly preceding the date of DOAC initiation) were excluded; in addition, patients were required to have a follow‐up period of at least one day to enable analysis. Specific inclusion and exclusion criteria are detailed in supplementary Table S1.

### Drug exposure and outcomes

Exposure to specific DOACs was defined as either yes or no. Patients initiating treatment with one drug were compared to patients initiating treatment with any of the other drugs; due to the censoring of patients at time of first treatment discontinuation and/or switch between drugs, all patients were continuously exposed to their index drug throughout their respective follow‐up periods. By focusing solely on the first treatment episode, differences in discontinuation rates and persistence to treatment between the different DOACs have been taken into account.

Treatment outcomes have been divided into two separate categories: effectiveness outcomes and safety outcomes. The primary effectiveness outcomes comprised stroke – all stroke (a composite of ischaemic and haemorrhagic stroke), as well as ischaemic stroke separately – systemic embolism, and death due to cardiovascular reasons as main clinical endpoints, comparable to the pivotal clinical trials. Also similar to the trials, secondary outcomes were pulmonary embolism, transient ischaemic attack, myocardial infarction, and all‐cause mortality; as well as composite effectiveness outcomes comprising (a) ischaemic stroke and systemic embolism; and (b) all stroke, systemic embolism and transient ischaemic attack. Safety outcomes all related to bleeding events, categorized as either major or non‐major/minor bleeding in the trials. Due to data availability, bleeding outcomes in this study have been divided into haemorrhagic stroke (comprising both intracranial and subarachnoid haemorrhages); other major bleeds, including other non‐traumatic intracranial haemorrhages and haemorrhages occurring within the chest or the respiratory or urinary tracts; and gastrointestinal bleeding. A composite outcome of all bleeds – comprising haemorrhagic stroke, other major bleeds and gastrointestinal bleeding – has been added in order to evaluate the overall risk of bleeding. ICD‐10 codes used to identify clinical endpoints are listed in supplementary Table S2.

Patients were censored after a first recorded event; however, all outcomes were analysed separately, meaning that while patients could have been subject to two different outcomes (e.g. a stroke and a major bleed), repeat events (i.e. a second stroke after having already suffered a stroke since initiating DOAC treatment) were not included in the analysis. More specifically, this means that a patient with both a stroke and a major bleed was included in the stroke analysis up to the time of first stroke, and in the major bleed analysis up to the time of first major bleed; if the stroke came first, the major bleed analysis was not censored at the time of stroke. The time to event was calculated based on the date of first prescription and date of hospital admission or date of death, as applicable.

### Statistical analysis

Crude incidence rates have been calculated and expressed as events per 100 person‐years. Statistical analysis of treatment outcomes has been conducted using multivariate Cox proportional hazard models, with time to event measured in days. The main exploratory variable was a categorical variable with three levels, indicating drug exposure (dabigatran, rivaroxaban or apixaban; no patients have initiated edoxaban treatment during the study period); binary variables (0 = no, 1 = yes) indicated the occurrence of an outcome of interest. A subgroup analysis – comprising patients with no observed changes in daily dose during the treatment period – has been performed to remove potential influences of dosage changes.

Variables used as covariates were chosen based on differences in patients' baseline characteristics, and comprised socio‐demographic factors (sex, age at time of first prescription, level of deprivation 27) as well as markers for comorbidities (Charlson score 28), stroke and bleeding risks (CHA_2_DS_2_‐VASc 29 and HAS‐BLED score 30, respectively), and polypharmacy (being treated with five or more drugs concomitantly). In order to account for observed geographical variations in prescribing patterns across Scotland, two additional variables – describing patients' place of residence in terms of Health Board and urban/rural classification 31 – have been included. All covariates were fixed at baseline; details regarding the calculation of the CHA_2_DS_2_‐VASc and HAS‐BLED scores can be found in supplementary Table S3.

All analyses were conducted using the R software, version 3.5.1 32.

### Ethical approval

Ethical approval was not required as only non‐identifiable routine data were used for this study, and no patient identifiers were available to the study team. However, use of the data has been approved by the appropriate Public Benefit and Privacy Panel for Health and Social Care 33. The data were hosted and managed by the NHS Scotland National Safe Haven 34.

## Results

A total of 18 456 patients with a diagnosis of AF, confirmed in secondary care, initiated treatment with dabigatran, rivaroxaban or apixaban during the study period. After excluding patients with valvular heart disease/heart valve replacement (*n* = 3077) or either a VTE (*n* = 679) or hip or knee replacement surgery (*n* = 220) 6 months prior to their first DOAC prescription, 14 634 patients remained; of these, a further 57 patients were excluded either because they have been treated with more than one DOAC simultaneously (*n* = 34), and due to having had less than one day of follow‐up (*n* = 23). Of the 14 577 patients included in the analysis, 6200 (42.5%) were treated with apixaban, 1112 (7.6%) with dabigatran and 7265 (49.8%) with rivaroxaban. For baseline characteristics, see Table 1.

**Table 1:** Patients' baseline characteristics, by drug prescribed

	Apixaban (*n* = 6200)	Dabigatran (*n* = 1112)	**Rivaroxaban (*n* = 7265)**	***P*‐value** a
**Calendar year of first prescription (%)**				
**2011**	0	48 (4.3)	0	
**2012**	0	350 (31.5)	439 (6.0)	
**2013**	341 (5.5)	356 (32.0)	1457 (20.1)	
**2014**	1950 (31.5)	246 (22.1)	2497 (34.4)	
**2015**	3909 (63.0)	112 (10.1)	2872 (39.5)	
**Median time of follow‐up [days] (IQR)**	188 (74–353)	216 (65–625.5)	243 (94–505)	<0.001
**Female (%)**	2885 (46.5)	410 (36.9)	3321 (45.7)	<0.001
**Mean age first prescription [years] (SD)**	73.7 (11.5)	71.1 (12.0)	74.8 (11.0)	<0.001
**<65 years (%)**	1224 (19.7)	299 (26.9)	1190 (16.4)	
**65–74 years (%)**	1696 (27.4)	342 (30.8)	2005 (27.6)	
**75 years and older (%)**	3280 (52.9)	471 (42.4)	4070 (56.0)	
**Mean Charlson score (SD)**	1.4 (1.7)	1.1 (1.5)	1.3 (1.7)	<0.001
**Mean CHA**_**2**_ **DS**_**2**_ **‐VASc score (SD)**	2.9 (1.7)	2.5 (1.8)	3.0 (1.7)	<0.001
**Comorbidities as included in CHA**_**2**_ **DS**_**2**_ **‐VASc score (%)** b				
**Congestive heart failure**	918 (14.8)	126 (11.3)	1009 (13.9)	0.007
**Hypertension**	2244 (36.2)	384 (34.5)	2722 (37.5)	0.092
**Diabetes mellitus**	952 (15.4)	149 (13.4)	1115 (15.3)	0.219
**Prior stroke/TIA**	908 (14.6)	137 (12.3)	1040 (14.3)	0.125
**Vascular disease**	1068 (17.2)	161 (14.5)	1339 (18.4)	0.003
**Mean HAS‐BLED score (SD)**	2.1 (1.2)	1.9 (1.2)	2.0 (1.2)	<0.001
**Conditions as included in HAS‐BLED score in addition to hypertension and prior stroke (%)** b				
**Renal disease**	947 (15.3)	97 (8.7)	1050 (14.5)	<0.001
**Liver disease**	13 (0.2)	<5	18 (0.2)	0.564
**Prior major bleeding**	587 (9.5)	117 (10.5)	673 (9.3)	0.409
**Medication usage** c	3153 (50.9)	514 (46.2)	3007 (41.4)	<0.001
**Alcohol usage**	275 (4.4)	55 (4.9)	266 (3.7)	0.025
**Polypharmacy (%)** d ^**,**^ e	5377 (86.7)	919 (82.6)	6417 (88.3)	<0.001
**Concomitant medication (%)** e				
**VKA**	1810 (29.2)	492 (44.2)	3190 (43.9)	<0.001
**Antiplatelet drugs**	2891 (46.6)	464 (41.7)	2762 (38.0)	<0.001
**NSAIDs**	471 (7.6)	92 (8.3)	460 (6.3)	0.004
**Digoxin**	1471 (23.7)	286 (25.7)	1739 (23.9)	0.355
**Beta‐blocker**	4353 (70.2)	700 (62.9)	4814 (66.3)	<0.001
**Anti‐diabetic drugs**	812 (13.1)	135 (12.1)	985 (13.6)	0.383
**Insulin**	216 (3.5)	36 (3.2)	292 (4.0)	0.175
**Statins**	3413 (55.0)	560 (50.4)	3908 (53.8)	0.012

IQR, interquartile range; NSAID, non‐steroidal anti‐inflammatory drug; SD, standard deviation; TIA, transient ischaemic attack; VKA, vitamin K antagonist (acenocoumarol, phenindione, warfarin sodium)

a Chi‐square or ANOVA comparing all groups

b Definitions are provided in supplementary Table S3

c Medication predisposing to bleeding, assessed during the 6‐month period prior to the first prescription (comprises anti‐platelet drugs and NSAIDs)

d Taking five or more different drugs concomitantly

e During a 6‐month period directly preceding initiation of DOAC treatment

During the study period, 192 ischaemic strokes, 18 systemic embolisms, and 613 deaths due to cardiovascular reasons occurred, in addition to 54 haemorrhagic strokes, 229 gastrointestinal bleeds, and 368 other bleeding events; crude incidence rates by drug prescribed are presented in Table 2.

**Table 2:** Crude incidence rates for all outcomes except composites, by drug prescribed

	**Apixaban (*n* = 6200)**		**Dabigatran (*n* = 1112)**		**Rivaroxaban (*n* = 7265)**	
	**No. of events**	**IR/100 PY**	**No. of events**	**IR/100 PY**	**No. of events**	**IR/100 PY**
**Ischaemic stroke**	67	1.66	19	1.62	106	1.61
**Systemic embolism**	5	0.12	2	0.17	11	0.17
**Death, cardiovascular**	196	4.83	39	3.30	378	5.70
**Pulmonary embolism**	7	0.17	0	0.0	29	0.44
**Transient ischaemic attack**	23	0.57	4	0.34	40	0.61
**Myocardial infarction**	77	1.91	7	0.59	57	0.86
**All‐cause mortality**	305	7.51	61	5.16	622	9.38
**Haemorrhagic stroke**	16	0.39	3	0.25	35	0.53
**Gastrointestinal bleeding**	69	1.71	22	1.88	138	2.10
**Other major bleeds**	104	2.59	23	1.97	241	3.71

IR, incidence rate; PY, person years

### Clinical effectiveness

**Figure 1:**
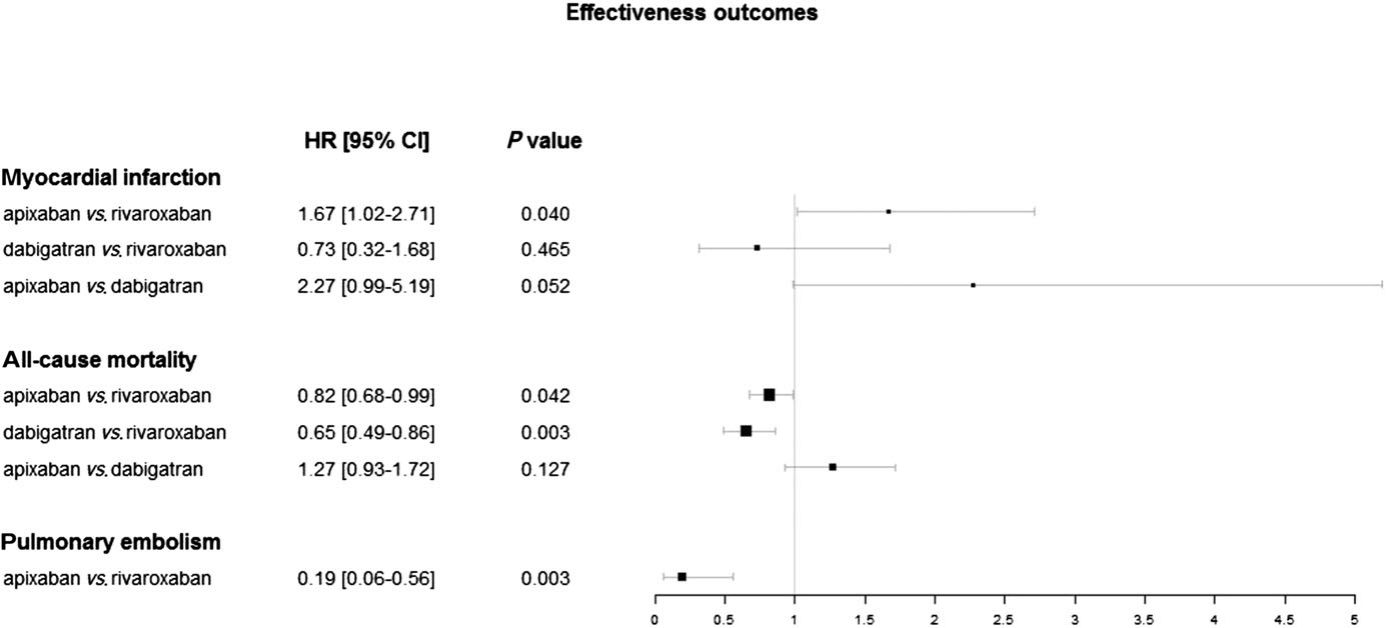
Adjusted hazard ratios and 95% confidence intervals for comparions of secondary effectiveness outcomes. CI, confidence interval; HR, hazard ratio

No differences between the DOACs were observed with respect to either primary effectiveness outcomes or the composite outcomes; there were, however, differences between drugs regarding three of the secondary effectiveness outcomes. Patients using rivaroxaban were 5.27 times [95% CI 1.79–15.53] more likely than patients being treated with apixaban to experience a pulmonary embolism; in contrast, apixaban patients were more likely to have a myocardial infarction than patients using rivaroxaban, with a hazard ratio of 1.67 [95% CI 1.02–2.71]. The use of rivaroxaban was associated with higher risks of all‐cause mortality as compared to both apixaban and dabigatran (apixaban: HR 0.82 [95% CI 0.68–0.99], dabigatran: HR 0.65 [95% CI 0.49–0.86]). See Table 3 for details; hazard ratios and 95% confidence intervals for comparisons with regard to secondary effectiveness outcomes are shown in Figure 1. Results are also presented in supplementary Table S4.

**Table 3:** Hazard ratios with 95% confidence intervals, multivariate models

**Reference: rivaroxaban**	**Apixaban**	***P*‐value**	**Dabigatran**	***P*‐value**
**Ischaemic stroke**	1.06 [0.68–1.64]	0.808	1.18 [0.70–2.01]	0.535
**All stroke**	0.89 [0.60–1.31]	0.557	1.01 [0.62–1.64]	0.981
**Systemic embolism**	0.59 [0.15–2.26]	0.441	1.54 [0.31–7.60]	0.596
**Death, cardiovascular**	0.92 [0.72–1.17]	0.476	0.71 [0.50–1.02]	0.065
**Pulmonary embolism**	0.19 [0.06–0.56]	0.003	n/a	
**Transient ischaemic attack**	0.75 [0.38–1.49]	0.413	0.63 [0.22–1.81]	0.390
**Myocardial infarction**	1.67 [1.02–2.71]	0.040	0.73 [0.32–1.68]	0.465
**All‐cause mortality**	0.82 [0.68–0.99]	0.042	0.65 [0.49–0.86]	0.003
**Composite A** a	0.98 [0.65–1.49]	0.941	1.18 [0.71–1.96]	0.517
**Composite B** b	0.85 [0.61–1.19]	0.340	0.98 [0.64–1.51]	0.936
**Haemorrhagic stroke**	0.53 [0.24–1.15]	0.106	0.43 [0.12–1.53]	0.194
**Gastrointestinal bleeding**	0.68 [0.46–0.99]	0.045	1.03 [0.63–1.68]	0.907
**Other major bleeds**	0.67 [0.49–0.91]	0.010	0.63 [0.40–0.99]	0.045
**All bleeds (composite)**	0.66 [0.52–0.83]	<0.001	0.74 [0.54–1.02]	0.067

a Ischaemic stroke + systemic embolism

b All stroke + transient ischaemic attack + systemic embolism

### Safety

**Figure 2:**
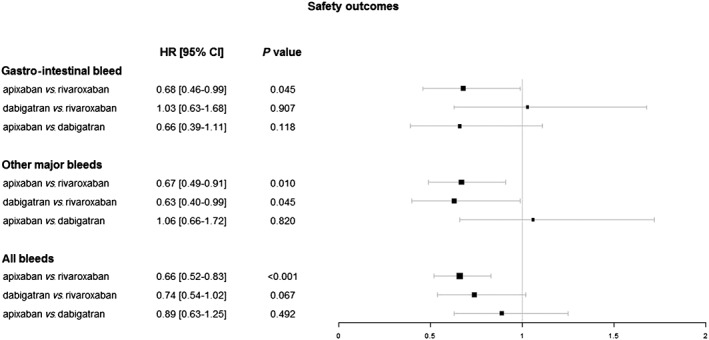
Adjusted hazard ratios and 95% confidence intervals for comparions of safety outcomes. CI, confidence interval; HR, hazard ratio

There were no significant differences between DOACs regarding haemorrhagic stroke, but there were with respect to gastrointestinal and other bleeds, as well as the composite outcome of all bleeding. Gastrointestinal bleeds as well as other major bleeds were 1.48 [95% CI 1.01–2.16] and 1.50 [95% CI 1.10–2.03] times more likely among patients using rivaroxaban than among apixaban patients, respectively; the overall bleeding risk – including all bleeding events – was 52% higher among patients being treated with rivaroxaban than among patients receiving apixaban (HR 1.52, 95% CI 1.21–1.92). While there were no differences between dabigatran and both apixaban and rivaroxaban with regard to gastrointestinal bleeds, the risk of other major bleeds was higher among rivaroxaban patients as compared to dabigatran (HR 1.58.95% CI 1.01–2.48). For details, see Table 3; comparisons of hazard ratios and 95% confidence intervals relating to safety outcomes are shown in Figure 2. Results are also presented in supplementary Table S5.

### Subgroup analysis

A total of 1066 patients (7.3%) experienced dosage changes during the treatment period; while 645 patients (4.4%) received reduced doses as compared to their initial dose, 384 patients (2.6%) increased dosage during the follow‐up period, and 37 patients (0.3%) were subject to more than one dosage change.

After excluding these patients, the majority of results were consistent with the main analysis: no differences were observed between the DOACs regarding ischaemic/all stroke, systemic embolism, transient ischaemic attack, composite effectiveness outcomes or haemorrhagic stroke, while findings indicated differences in the risks of pulmonary embolism, all‐cause mortality and overall bleeding.

Results differed, however, with respect to the other outcomes. In the subgroup analysis, no differences were found in the risks of myocardial infarction and gastrointestinal bleeding; or between rivaroxaban and dabigatran regarding other major bleeds. In contrast, a higher risk of cardiovascular death was observed in rivaroxaban patients as compared to patients being treated with dabigatran (HR 1.54 [95% CI 1.05–2.28]). Details are presented in supplementary Table S6.

## Discussion

This was the first study in Scotland using linked data to analyse the clinical effectiveness and safety of DOAC treatment in patients with AF, and one of a growing number of international observational studies directly comparing the individual DOACs – either focusing on safety 35, 36, or analysing both safety and effectiveness 37, 38, 39, 40, 41. As one of the few nationwide studies conducted outside the US thus far, it provides exceptional population coverage. In addition, it offers a comprehensive overview of DOAC treatment outcomes by including all drugs available during the study period, as well as evaluating both their clinical effectiveness and safety.

The main study findings are, by and large, comparable to previously published results: although bleeding risks differ between individual drugs, all DOACs are similarly effective in preventing strokes and systemic embolisms. Nevertheless, a number of unexpected results emerged from this study: first, rivaroxaban seems to be associated with an increased risk of all‐cause mortality as compared to apixaban and dabigatran; second, patients treated with apixaban appeared to have a higher risk of experiencing a myocardial infarction than patients treated with rivaroxaban; and third, apixaban might potentially be more effective in preventing pulmonary embolisms than rivaroxaban.

In this study, no significant differences between DOACs were found in the risks of strokes, systemic embolism, transient ischaemic attacks or combinations thereof – in line with the majority of previously published research. Other observational studies also found no differences in the risk of stroke between dabigatran and rivaroxaban 38, the combination of stroke/systemic embolism between apixaban and dabigatran 37 or between any of the DOACs 41, or a composite outcome of stroke, systemic embolism and death across all DOACs 39, principally confirming conclusions made based on indirect comparisons between DOACs using clinical trial data 12, 13, 14. Unlike the results with regard to these primary outcomes, which have shown high agreement across studies, findings relating to secondary outcomes were nevertheless varied. The risk of all‐cause mortality, for instance, was 55% higher among rivaroxaban patients than among dabigatran patients in this study (albeit with a relatively wide confidence interval around the HR), and comparable findings have been reported before: 43% in a nationwide Danish study 38; and 44% in an observational study from Taiwan 40. Results with respect to comparative risks of myocardial infarction differed, however, between studies. While our study found a higher risk among patients using apixaban as compared to rivaroxaban, other observational studies found no differences or did not report them 38, 40, while indirect comparisons – based on clinical trials data – suggested higher risks in dabigatran in comparison to apixaban and/or rivaroxaban 12, 13. Moreover, the risks of pulmonary embolisms have rarely been analysed; an indirect comparison of apixaban and dabigatran found no difference between the DOACs 12.

Due to the disparity of secondary effectiveness outcomes used across studies – both in terms of what has been used, and how it has been defined – and the overall still limited number of studies directly comparing DOACs thus far, there is only restricted potential to compare results. Consequently, any reasons for observed differences in the risks of all‐cause mortality, myocardial infarction and especially pulmonary embolism are difficult to discern. A suggested explanation for differences in all‐cause mortality was selective prescribing for rivaroxaban based on the relatively higher‐risk population included in the ROCKET‐AF trial 38; due to our study design and the data available for analysis, unobserved differences in patient characteristics, linked to the prescribing or non‐prescribing of specific drugs, were most likely contributing factors. Similarly, considering the divergence of findings between the overall study population and the subgroup analysis comprising patients not subject to dosage changes, observed differences in myocardial infarction might have been due to differences in patient characteristics; however, as reasons for dosage changes were unknown, additional interpretations of results would be speculative. Furthermore, pulmonary embolisms were very rare events during the study period (*n* = 36), and the treatment groups in our study were quite different in size, with considerably fewer patients having initiated treatment with dabigatran (*n* = 1112) than with either apixaban (*n* = 6200) or rivaroxaban (*n* = 7265) – resulting in no pulmonary embolism having occurred among dabigatran patients during the study period.

While differences across DOACs with respect to the risk of haemorrhagic strokes were not significant in this study, the risks of gastrointestinal bleeding, other major non‐fatal bleeds, and bleeding overall were 48%, 50% and 52% higher among patients using rivaroxaban than among apixaban patients, respectively; there were, however, no differences between apixaban and dabigatran in any of the safety outcomes, and observed differences between rivaroxaban and dabigatran were inconsistent. Similar findings have been reported before, albeit with some variation in individual results. Several previous studies have highlighted differences in bleeding risks between rivaroxaban and apixaban, with risks for gastrointestinal bleeding 35, 37, 39, other major bleeding 35, 36, 41 and overall bleeding risks 37, 39 all significantly higher among rivaroxaban patients. However, other studies also reported bleeding risks to be higher among rivaroxaban patients than among patients using dabigatran 38, 39, 40, 41, a finding that was observed for other bleeds but not gastrointestinal bleeding in our study; this could, however, be due to different definitions of bleeding being used. As in this study, several other studies reported no differences in bleeding events between apixaban and dabigatran 36, 37, 39; most other studies also did not find any differences with regard to haemorrhagic stroke.

### Strengths and limitations

This study has several strengths. As access to health care in Scotland is universal, electronic patient records are available for the entire population; records can be easily and reliably linked due to the presence of a unique patient identifier; and the validity and accuracy of the data stored in these records have been established 21, 42, 43.

Nevertheless, there are also some limitations to consider. First of all, patients to be included in the study have been identified in secondary care only, meaning that patients diagnosed and treated exclusively in primary care – potentially with less severe representations of AF – were not captured. The impact of not including these patients on hazard ratios when comparing DOACs with each other was, however, most likely small. As current treatment guidelines do not recommend specific DOACs based on varying levels of disease severity, the non‐inclusion of patients should have been independent of the drug they received when initiating DOAC treatment. Second, as the data used for analysis were not collected specifically for the purpose of this study but stemmed from a limited set of administrative records, not all desirable information was available; identification of endpoints, for example, had to rely on ICD‐10 codes available from hospital discharge and death records, with possible implications on the accuracy of findings – particularly regarding bleeding events. While this might potentially have resulted in an underestimation of the bleeding risks associated with DOAC use, its impact on hazard ratios is likely limited. Furthermore, as information regarding indication for prescribing was not available, patients might have been included in this study who had AF but were treated with DOACs for other reasons; however, by excluding patients with recorded diagnoses of alternative indications for treatment, this should have been kept to a minimum, thus limiting the effect on results. Third, adjustment for covariates was done with covariates scored at baseline only, even though the use of medication potentially impacting bleeding risks (such as NSAIDs) while being treated with DOACs could have affected the accuracy of findings. As NSAIDs as well as aspirin are available over‐the‐counter without a prescription in Scotland, full adjustments for use of these drugs was, however, not feasible. And finally, the analysis assumed that effects of the covariates were consistent across all DOACs; this might not necessarily be the case, with potential implications on the accuracy of findings. In addition, as with all observational studies, associations between exposure and outcomes might not have been causal. While efforts have been made to reduce the impact of confounding factors as far as possible, unmeasured confounding could, nevertheless, have influenced the findings.

## Conclusion

In general, this study has mostly confirmed what has been reported before: all DOACs were similarly effective in preventing strokes and systemic embolisms in patients with atrial fibrillation, but the bleeding profiles differ slightly between drugs – with rivaroxaban showing the highest overall risk of bleeding. Nevertheless, the study also indicated potentially vital differences in the risks of all‐cause mortality and myocardial infarction between the DOACs, and provided a new signal with regard to pulmonary embolism, warranting further research.

## Competing Interests

O.W. has received funding as a consultant from Bayer to provide methodological advice on evidence synthesis of randomized controlled trial (RCT) and non‐RCT data relating to DOACs. All other authors have no competing interests to declare.

*We acknowledge the funding support from The Farr Institute @ Scotland. The Farr Institute @ Scotland is supported by a 10‐funder consortium:* *Arthritis Research UK* *,* *the British Heart Foundation* *,* *Cancer Research UK* *,* *the Economic and Social Research Council* *,* *the Engineering and Physical Science Research Council* *,* *the Medical Research Council* *,* *the National Institute of Health Research* *,* *the National Institute for Social Care and Health Research* *(Welsh Assembly Government), the Chief Scientist Office (Scottish Government Health Directorates), the* *Wellcome Trust* *(MRC Grant No:* MR/K007017/1*). We thank the members of the Information Services Division, National Services Scotland, for their support*.

## Contributors

All authors contributed to the study concept and design. T.M. performed the data analysis and drafted the paper. C.R., O.W. and M.B. contributed to the interpretation of results. All authors were involved in critically revising the text, and all authors read and approved the final version of the manuscript. M.B. was the Principal investigator.

## Supporting information


**Table S1** Study population inclusion and exclusion criteria
**Table S2** Diagnostic codes used to identify study endpoints
**Table S3** Methods and codes used to calculate stroke and bleeding risk scores
**Table S4** Hazard ratios with 95% confidence intervals for significant associations between drug and effectiveness outcome, all comparisons
**Table S5** Hazard ratios with 95% confidence intervals for significant associations between drugs and safety outcomes, all comparisons
**Table S6** Hazard ratios with 95% confidence intervals, multivariate models – subgroup analysis
