# Supplementary material for: Comparative safety and effectiveness of direct oral anticoagulants in patients with atrial fibrillation in clinical practice in Scotland
Source: Br J Clin Pharmacol. 2018 Dec 18;85(2):422–31. doi: 10.1111/bcp.13814 (PMC6339970; doi:10.1111/bcp.13814)
Supplement: Supplementary file 1 — Table S1 Study population inclusion and exclusion criteria Table S2 Diagnostic codes used to identify study endpoints Table S3 Methods and codes used to calculate stroke and bleeding risk scores Table S4 Hazard ratios with 95% confidence intervals for significant associations between drug and effectiveness outcome, all comparisons Table S5 Hazard ratios with 95% confidence intervals for significant associations between drugs and safety outcomes, all comparisons Table S6 Hazard ratios with 95% confidence intervals, multivariate models – subgroup analysis [file BCP-85-422-s001.docx]

**Supporting Information**

Table S1: Study population inclusion and exclusion criteria

| Study population selection | Method of identification |
| --- | --- |
| 1. Inclusion criteria (sources: SMR01, PIS) | |
| *Atrial fibrillation (AF)* | **ICD-10 code** I48 |
| *Direct oral anticoagulant (DOAC)* | **BNF name** apixaban, dabigatran etexilate, rivaroxaban, edoxaban |
| 1. Exclusion criteria (sources: SMR00, SMR01, PIS) | |
| *Rivaroxaban 2.5mg †* | **BNF name** rivaroxaban & **BNF drug strength** 2.5 |
| *Index prescription prior to drug’s SMC approval for the indication of stroke prevention in patients with AF ‡* | **Date of first recorded DOAC prescription prior to**  Dabigatran: 05.08.2011  Rivaroxaban: 13.01.2012  Apixaban: 11.01.2013  Edoxaban: 09.10.2015 |
| *Valvular disease or heart valve replacement* | **ICD-10 codes** I05, I06, I08, I34, I35, Q23, Z95.2 – Z95.4  **OPCS-4 codes** K25.2 – K25.4, K26.2 – K26.4, K29.2 – K29.4 |
| *VTE 6 months prior to first prescription* | **ICD-10 codes** I26, I63.3, I67.6, I80.1 – I80.9, I81, I82.2 – I82.9 |
| *Hip or knee replacement surgery 6 months prior to first prescription* | **OPCS-4 codes** O18, W37 – W42, W93 – W95 |

AF – atrial fibrillation; BNF – British National Formulary; DOAC – direct oral anticoagulant; ICD-10 – International Classification of Disease, 10^th^ edition; OPCS-4 – Office of Population Censuses and Surveys procedural codes, 4^th^ revision; PIS – Prescribing Information Service; SMC – Scottish Medicines Consortium; SMR – Scottish Morbidity Records; VTE – venous thromboembolism

† exclusively indicated for patients with acute coronary syndrome

‡ treatment prior to official approval might indicate alternative reasons for treatment

Table S2: Diagnostic codes used to identify study endpoints

| Endpoint/outcomes | ICD-10 codes |
| --- | --- |
| 1. Clinical effectiveness (sources: SMR00, SMR01, NRS) | |
| *Stroke, all* | I60, I61, I63, I64 |
| *Ischaemic stroke* | I63, I64 |
| *Transient ischaemic attack* | G45.8, G45.9 |
| *Systemic embolism* | I74 |
| *Pulmonary embolism* | I26 |
| *Myocardial infarction* | I21, I22 |
| *Death, cardiovascular* | I11, I13, I20 – I26, I46, I47, I49, I50, I60, I61, I63, I64, I67, I73, I74 |
| 1. Safety (sources: SMR00, SMR01) | |
| *Haemorrhagic stroke* | I60, I61 |
| *Other major bleeds* | D62, H11.3, H35.6, H43.1, I62, J94.2, N02, N95.0, R04, R31, R58 |
| *Gastro-intestinal bleeds* | K25.0, K25.2, K25.4, K25.6, K26.0, K26.2, K26.4, K26.6, K27.0, K27.2, K27.4, K27.6, K28.0, K28.2, K28.4, K28.6, K29.0, K62.5, K92.0 – K92.2 |

ICD-10 – International Classification of Disease, 10^th^ edition; NRS – National Records of Scotland; SMR – Scottish Morbidity Records

Table S3: Methods and codes used to calculate stroke and bleeding risk scores

| Score element | Method of identification |
| --- | --- |
| 1. CHA_2_DS_2_-VASc score (sources: SMR00, SMR01) | |
| *Congestive heart failure* | **ICD-10 codes** I11.0, I13.0, I13.2, I50 |
| *Hypertension* | **ICD-10 codes** I10 – I15 |
| *Diabetes mellitus* | **ICD-10 codes** E10, E11, E13, E14, G59.0, G63.2, H28.0, H36.0, M14.2, N08.3, O24.0, O24.1, O24.3 |
| *Prior stroke/TIA* | **ICD-10 codes** I63, I64, G45.8, G45.9, G46.3 – G46.7 |
| *Vascular disease* | **ICD-10 codes** I20 – I22, I70, I73.1, I73.8, I73.9, I74 |
| 1. HAS-BLED score (sources: SMR00, SMR01, PIS) † | |
| *Hypertension* | **ICD-10 codes** I10 – I15 |
| *Renal disease* | **ICD-10 codes** I12, I13, N00 – N05, N07, N11, N14, N17 – N19, Q61 |
| *Liver disease* | **ICD-10 codes** B15.0, B16.0, B16.2, B19.0, K70.4, K72, K76.5 – K76.7 |
| *Prior stroke/TIA* | **ICD-10 codes** I63, I64, G45.8, G45.9, G46.3 – G46.7 |
| *Prior major bleeding* | **ICD-10 codes** D62, H11.3, H35.6, H43.1, I60 – I62, J94.2, K25.0, K25.5, K25.4, K25.6, K26.0, K26.2, K26.4, K26.6, K27.0, K27.2, K27.4, K27.6, K28.0, K28.2, K28.4, K28.6, K29.0, K62.5, K92.0 – K92.2, N02, N95.0, R04, R31, R58 |
| *Medication usage predisposing to bleeding* | **BNF codes** 02.09 (antiplatelet drugs), 10.01.01 (NSAIDs) |
| *Alcohol usage* | **ICD-10 codes** E52, F10, G31.2, G62.1, G72.1, I42.6, K29.2, K70, K86.0, O35.4, T51, Z71.4, Z72.1  **BNF code** 04.10.01 (drugs used in alcohol dependence) |

BNF – British National Formulary; ICD-10 – International Classification of Disease, 10^th^ edition; NSAID – non-steroidal anti-inflammatory drug; PIS – Prescribing Information System; SMR – Scottish Morbidity Records; TIA – transient ischaemic attack

† does not include points for labile INR as this information was not available for this study

*Table S4: Hazard ratios with 95% confidence intervals for significant associations between drug and effectiveness outcome, all comparisons*

|  | Pulmonary embolism | | Myocardial infarction | | All-cause mortality | |
| --- | --- | --- | --- | --- | --- | --- |
|  | ***Hazard ratio*** | ***p-value*** | ***Hazard ratio*** | ***p-value*** | ***Hazard ratio*** | ***p-value*** |
| *Apixaban* | 0.19  [0.06 – 0.56] | 0.003 | 1.67  [1.02 – 2.71] | 0.040 | 0.82  [0.68 – 0.99] | 0.042 |
| *Dabigatran* | n/a ‡ |  | 0.73  [0.32 – 1.68] | 0.465 | 0.65  [0.49 – 0.86] | 0.003 |
| *Rivaroxaban †* | 1 |  | 1 |  | 1 |  |
| *Apixaban* | n/a ‡ |  | 2.27  [0.99 – 5.19] | 0.052 | 1.27  [0.93 – 1.72] | 0.127 |
| *Dabigatran †* | n/a ‡ |  | 1 |  | 1 |  |
| *Rivaroxaban* | n/a ‡ |  | 1.36  [0.60 – 3.11] | 0.465 | 1.55  [1.16 – 2.05] | 0.003 |
| *Apixaban †* | 1 |  | 1 |  | 1 |  |
| *Dabigatran* | n/a ‡ |  | 0.44  [0.19 – 1.01] | 0.052 | 0.79  [0.58 – 1.07] | 0.127 |
| *Rivaroxaban* | 5.27  [1.79 – 15.53] | 0.003 | 0.60  [0.37 – 0.98] | 0.040 | 1.22  [1.01 – 1.47] | 0.042 |

† reference drug;

‡ no events observed

*Table S5: Hazard ratios with 95% confidence intervals for significant associations between drugs and safety outcomes, all comparisons*

|  | Gastro-intestinal bleed | | Other major bleed | | All bleeds ‡ | |
| --- | --- | --- | --- | --- | --- | --- |
|  | ***Hazard ratio*** | ***p-value*** | ***Hazard ratio*** | ***p-value*** | ***Hazard ratio*** | ***p-value*** |
| *Apixaban* | 0.68  [0.46 – 0.99] | 0.045 | 0.67  [0.49 – 0.91] | 0.010 | 0.66  [0.52 – 0.83] | <0.001 |
| *Dabigatran* | 1.03  [0.63 – 1.68] | 0.907 | 0.63  [0.40 – 0.99] | 0.045 | 0.74  [0.54 – 1.02] | 0.067 |
| *Rivaroxaban †* | 1 |  | 1 |  | 1 |  |
| *Apixaban* | 0.66  [0.39 – 1.11] | 0.118 | 1.06  [0.65 – 1.72] | 0.820 | 0.89  [0.63 – 1.25] | 0.492 |
| *Dabigatran †* | 1 |  | 1 |  | 1 |  |
| *Rivaroxaban* | 0.97  [0.59 – 1.59] | 0.907 | 1.58  [1.01 – 2.48] | 0.045 | 1.35  [0.98 – 1.86] | 0.067 |
| *Apixaban †* | 1 |  | 1 |  | 1 |  |
| *Dabigatran* | 1.52  [0.90 – 2.57] | 0.118 | 0.95  [0.58 – 1.54] | 0.820 | 1.13  [0.80 – 1.60] | 0.492 |
| *Rivaroxaban* | 1.48  [1.01 – 2.16] | 0.045 | 1.50  [1.10 – 2.03] | 0.010 | 1.52  [1.21 – 1.92] | <0.001 |

† reference drug

‡ comprises haemorrhagic stroke, gastro-intestinal bleeds, and other major bleeds

*Table S6: Hazard ratios with 95% confidence intervals, multivariate models – subgroup analysis*

| *Reference: rivaroxaban* | Apixaban | p-value | Dabigatran | p-value |
| --- | --- | --- | --- | --- |
| *Ischaemic stroke* | 1.05  [0.65 – 1.68] | 0.845 | 1.21  [0.68 – 2.15] | 0.514 |
| *All stroke* | 0.91  [0.60 – 1.37] | 0.642 | 1.06  [0.63 – 1.79] | 0.833 |
| *Systemic embolism* | 0.41  [0.09 – 1.76] | 0.229 | 1.68  [0.32 – 8.75] | 0.536 |
| *Death, cardiovascular* | 0.83  [0.64 – 1.08] | 0.162 | 0.65  [0.44 – 0.95] | 0.028 |
| *Pulmonary embolism* | 0.28  [0.09 – 0.84] | 0.023 | n/a |  |
| *Transient ischaemic attack* | 0.62  [0.29 – 1.35] | 0.229 | 0.84  [0.28 – 2.47] | 0.751 |
| *Myocardial infarction* | 1.38  [0.82 – 2.30] | 0.224 | 0.69  [0.28 – 1.68] | 0.415 |
| *All-cause mortality* | 0.78  [0.64 – 0.95] | 0.015 | 0.61  [0.45 – 0.82] | 0.001 |
| *Composite A †* | 0.95  [0.60 – 1.48] | 0.806 | 1.22  [0.71 – 2.10] | 0.473 |
| *Composite B ‡* | 0.83  [0.58 – 1.19] | 0.308 | 1.09  [0.69 – 1.73] | 0.703 |
| *Haemorrhagic stroke* | 0.60  [0.27 – 1.34] | 0.216 | 0.52  [0.14 – 1.84] | 0.307 |
| *Gastro-intestinal bleeding* | 0.70  [0.47 – 1.05] | 0.088 | 1.01  [0.59 – 1.71] | 0.979 |
| *Other major bleeds* | 0.69  [0.49 – 0.96] | 0.027 | 0.64  [0.39 – 1.05] | 0.077 |
| *All bleeds (composite)* | 0.67  [0.52 – 0.86] | 0.002 | 0.74  [0.53 – 1.05] | 0.097 |

† ischaemic stroke + systemic embolism

‡ all stroke + transient ischaemic attack + systemic embolism
